# Supplementary material for: Formation of ultralong DH regions through genomic rearrangement
Source: BMC Immunol. 2020 Jun 2;21:30. doi: 10.1186/s12865-020-00359-8 (PMC7265228; doi:10.1186/s12865-020-00359-8)

**Supplemental Figure 1.** Alignment of all bovine DH regions in clusters 2-4 was performed using Clustal Omega. (A) Percent identity matrix. (B) Sequence alignment. (C) Phylogenetic tree.

**A.**

#

#

# Percent Identity Matrix - created by Clustal2.1

#

#

**2-2 2-3 2-4 1-3 1-2 1-4 5-2 5-3 5-4 3-3 3-4 7-3 7-4 8-2 6-2 6-3 6-4**

1: IGHD2_2 100.00 100.00 100.00 36.11 37.04 40.74 52.78 52.78 52.78 48.61 48.61 50.70 50.70 52.78 50.70 49.30 49.30

2: IGHD2_3 100.00 100.00 100.00 36.11 37.04 40.74 52.78 52.78 52.78 48.61 48.61 50.70 50.70 52.78 50.70 49.30 49.30

3: IGHD2_4 100.00 100.00 100.00 36.11 37.04 40.74 52.78 52.78 52.78 48.61 48.61 50.70 50.70 52.78 50.70 49.30 49.30

4: IGHD1_3 36.11 36.11 36.11 100.00 97.70 98.85 39.80 40.82 40.82 43.48 43.48 37.07 36.07 41.62 47.87 46.81 46.81

5: IGHD1_2 37.04 37.04 37.04 97.70 100.00 98.85 55.17 55.17 55.17 60.00 60.00 55.88 55.88 49.41 60.00 58.00 58.00

6: IGHD1_4 40.74 40.74 40.74 98.85 98.85 100.00 58.62 58.62 58.62 63.33 63.33 58.82 58.82 50.59 62.00 60.00 60.00

7: IGHD5_2 52.78 52.78 52.78 39.80 55.17 58.62 100.00 97.96 97.96 71.43 71.43 74.16 73.68 72.45 78.08 76.71 76.71

8: IGHD5_3 52.78 52.78 52.78 40.82 55.17 58.62 97.96 100.00 100.00 72.53 72.53 73.03 72.63 71.43 79.45 78.08 78.08

9: IGHD5_4 52.78 52.78 52.78 40.82 55.17 58.62 97.96 100.00 100.00 72.53 72.53 73.03 72.63 71.43 79.45 78.08 78.08

10: IGHD3_3 48.61 48.61 48.61 43.48 60.00 63.33 71.43 72.53 72.53 100.00 100.00 78.05 78.41 79.35 87.84 85.14 85.14

11: IGHD3_4 48.61 48.61 48.61 43.48 60.00 63.33 71.43 72.53 72.53 100.00 100.00 78.05 78.41 79.35 87.84 85.14 85.14

12: IGHD7_3 50.70 50.70 50.70 37.07 55.88 58.82 74.16 73.03 73.03 78.05 78.05 100.00 100.00 84.55 95.71 95.71 95.71

13: IGHD7_4 50.70 50.70 50.70 36.07 55.88 58.82 73.68 72.63 72.63 78.41 78.41 100.00 100.00 84.50 95.71 95.71 95.71

14: IGHD8_2 52.78 52.78 52.78 41.62 49.41 50.59 72.45 71.43 71.43 79.35 79.35 84.55 84.50 100.00 89.47 89.47 89.47

15: IGHD6_2 50.70 50.70 50.70 47.87 60.00 62.00 78.08 79.45 79.45 87.84 87.84 95.71 95.71 89.47 100.00 96.49 96.49

16: IGHD6_3 49.30 49.30 49.30 46.81 58.00 60.00 76.71 78.08 78.08 85.14 85.14 95.71 95.71 89.47 96.49 100.00 100.00

17: IGHD6_4 49.30 49.30 49.30 46.81 58.00 60.00 76.71 78.08 78.08 85.14 85.14 95.71 95.71 89.47 96.49 100.00 100.00

**B.**

IGHD2_2 GCTTTTTGCCAAGGGCTCTACTGCGGT--------------------------------- 27

IGHD2_3 GCTTTTTGCCAAGGGCTCTACTGCGGT--------------------------------- 27

IGHD2_4 GCTTTTTGCCAAGGGCTCTACTGCGGT--------------------------------- 27

IGHD1_3 GGATTTTGAGGGTGTGCGTGTCACCGTGAGACTATCGTGATGATGGTTACTG-------- 52

IGHD1_2 GGATTTTGAGGGTGTGCGTGTCACCCTGAGAATATCGTGATGATGGTTACTG-------- 52

IGHD1_4 GGATTTTGAGGGTGTGCGTGTCACCGTGAGAATATCGTGATGATGGTTACTG-------- 52

IGHD5_2 GGTTTCTGATGCCGGCTGTGTTGTGGTGA------------------------------- 29

IGHD5_3 GGTTTCTGATGCCGGCTGTGTTGTGGTGA------------------------------- 29

IGHD5_4 GGTTTCTGATGCCGGCTGTGTTGTGGTGA------------------------------- 29

IGHD3_3 GGTTTCTGATGCCGGCTGTGTCACGGTGGT------------------------------ 30

IGHD3_4 GGTTTCTGATGCCGGCTGTGTCACGGTGGT------------------------------ 30

IGHD7_3 GGTTTTTGATGCCGGCTGTGTCACGG---------------------------------- 26

IGHD7_4 GGTTTTTGATGCCGGCTGTGTCACGG---------------------------------- 26

IGHD8_2 GGTTTCTGATGCCGGCTGTGTCACGGTGGT--AGTTGTCCTGATGGTTATAGTTATGGTT 58

IGHD6_2 GGTTTCTGATGCCGGCTGTGTCACGGTGGT--AGTTGTTATAGTGGTTATGGTTATGGTT 58

IGHD6_3 GGTTTCTGATGCCAGCTGTGTCACGGTGGT--AGTTGTTATAGTGGTTATGGTTATGGTT 58

IGHD6_4 GGTTTCTGATGCCAGCTGTGTCACGGTGGT--AGTTGTTATAGTGGTTATGGTTATGGTT 58

* ** ** *

IGHD2_2 ------------------------------------------------------------ 27

IGHD2_3 ------------------------------------------------------------ 27

IGHD2_4 ------------------------------------------------------------ 27

IGHD1_3 ----------------CTACACCCACAGTGACTCAGGCCCTGACATAAAGTCT------G 90

IGHD1_2 ----------------CTACACCCACAGTGACTCAGGCCCTGACATAAAGT--------- 87

IGHD1_4 ----------------CTACACCCACAGTGACTCAGGCCCTGACATAAAGT--------- 87

IGHD5_2 ------------------------------------------------------------ 29

IGHD5_3 ------------------------------------------------------------ 29

IGHD5_4 ------------------------------------------------------------ 29

IGHD3_3 ------------------------------------------------------------ 30

IGHD3_4 ------------------------------------------------------------ 30

IGHD7_3 -------------------------------------------TGGTAGTTATGGTGGTT 43

IGHD7_4 -------------------------------------------TGGTAGTTATGGTGGTT 43

IGHD8_2 ATGGTTGTGGTTATGGTTATGGTTGTAGTGGTTATGATTGTTATGGTTATGGTGGTTATG 118

IGHD6_2 GTGGTTATGGTT------------------------------------------------ 70

IGHD6_3 ATGGTTGTGGTT------------------------------------------------ 70

IGHD6_4 ATGGTTGTGGTT------------------------------------------------ 70

IGHD2_2 -----------------------------------------GTTACTATAGTGACCACCA 46

IGHD2_3 -----------------------------------------GTTACTATAGTGACCACCA 46

IGHD2_4 -----------------------------------------GTTACTATAGTGACCACCA 46

IGHD1_3 ACCCGCACAC-AGGTGTGGAGCTGGCCAATGCATCCCCAGGGGCACTGGGCTCCCAAGCA 149

IGHD1_2 ------------------------------------------------------------ 87

IGHD1_4 ------------------------------------------------------------ 87

IGHD5_2 -----------------TGATACGATAGGTGTGGTTGTAGTTATTGTAGTGTTGCTACCA 72

IGHD5_3 -----------------TGATACGATAGGTGTGGTTTTAGTTATTGTAGTGTTGCTACCA 72

IGHD5_4 -----------------TGATACGATAGGTGTGGTTTTAGTTATTGTAGTGTTGCTACCA 72

IGHD3_3 ------------------------ATTGTGGTAGCTATTGTGGTAGTTATTATGGTACCA 66

IGHD3_4 ------------------------ATTGTGGTAGCTATTGTGGTAGTTATTATGGTACCA 66

IGHD7_3 ATGGTTATGGTGGTTATGGTTGTTATGGTTATGGT------TATGGTTATGGTTATACCA 97

IGHD7_4 ATGGTTATGGTGGTTATGGTTGTTATGGTTATGGTTATGGTTATGGTTATGGTTATACCA 103

IGHD8_2 GTGGTTATGGTGGTTATGGTTATAGTAGTTATAGTTATAGTTATACTTACGAATATACCA 178

IGHD6_2 ------------------------------------------ATGGTTATGATTATACCA 88

IGHD6_3 ------------------------------------------ATGGTTATGGTTATACCA 88

IGHD6_4 ------------------------------------------ATGGTTATGGTTATACCA 88

IGHD2_2 CAGTGAGACATGGGGCAGCAAACCCT 72

IGHD2_3 CAGTGAGACATGGGGCAGCAAACCCT 72

IGHD2_4 CAGTGAGACATGGGGCAGCAAACCCT 72

IGHD1_3 AGGTGCCTATCCCCCCAACTCGGGAC 175

IGHD1_2 -------------------------- 87

IGHD1_4 -------------------------- 87

IGHD5_2 CAGTGATGCTCTCAGTGTCAGAAACC 98

IGHD5_3 CAGTGACGCTCTCAGTGTCAGAAACC 98

IGHD5_4 CAGTGACGCTCTCAGTGTCAGAAACC 98

IGHD3_3 CAGTGACACTGTCCAGGACAGAAACC 92

IGHD3_4 CAGTGACACTGTCCAGGACAGAAACC 92

IGHD7_3 CAGTGATACTCTCTGGGACAAAAACC 123

IGHD7_4 CAGTGATACTCTCTGGGACAAAAACC 129

IGHD8_2 CAGTGATACTCTCTGGGACAAAAACC 204

IGHD6_2 CAGTGACACTCTCTGGGACAAAAACC 114

IGHD6_3 CAGTGACACTCTCTGGGACAAAAACC 114

IGHD6_4 CAGTGACACTCTCTGGGACAAAAACC 114

**C.**

###
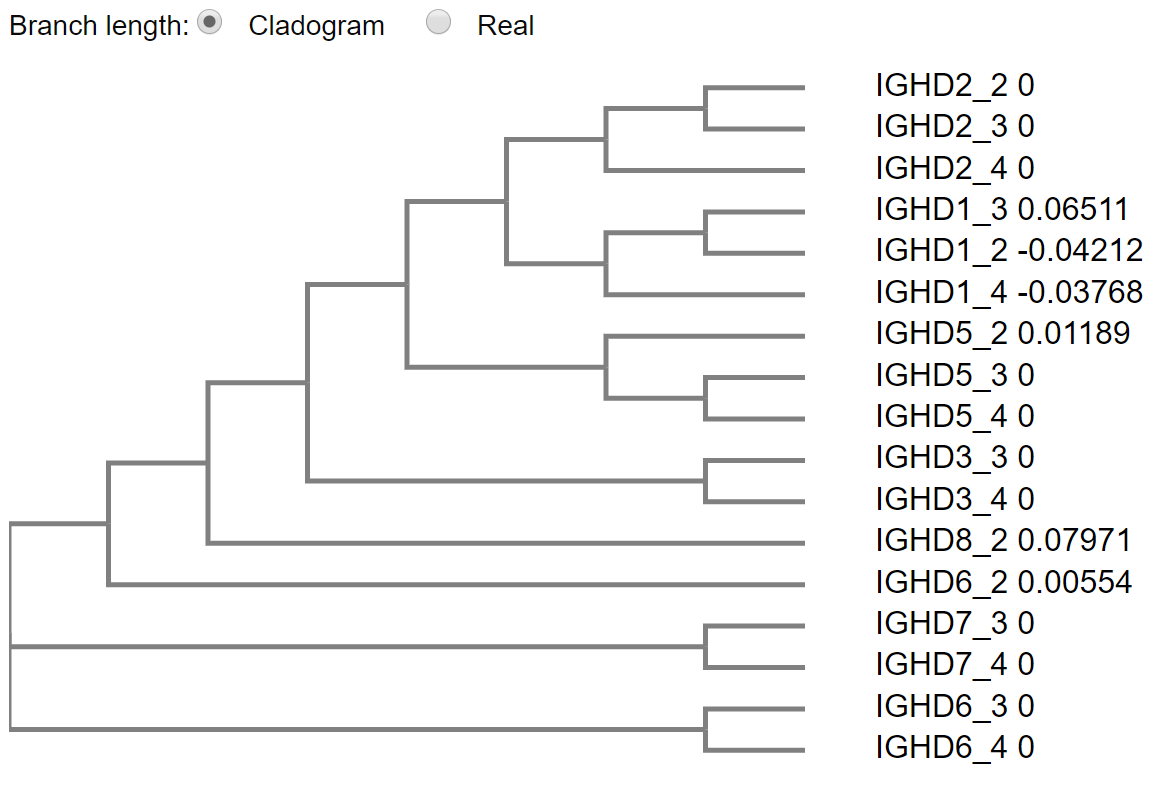

Supplement: Supplementary file 1 — Additional file 1: Figure S1. Alignment of Bos taurus DH regions. [file 12865_2020_359_MOESM1_ESM.docx]
