# Supplementary material for: Formation of ultralong DH regions through genomic rearrangement
Source: BMC Immunol. 2020 Jun 2;21:30. doi: 10.1186/s12865-020-00359-8 (PMC7265228; doi:10.1186/s12865-020-00359-8)
Supplement: Supplementary file 2 — Additional file 2: Figure S2. Alignment of DH clusters 2, 3, and 4. [file 12865_2020_359_MOESM2_ESM.docx]

**Supplemental Figure 2.** Global multiple sequence alignment of clusters 2-4 (above) using Webprank, or clusters 2 and 3 (below) using Stretcher. The DH regions are color coded as described in the Key. Note that IGHD5-2 is forced to align with IGHD3 in the global alignment. Supplemental Figures 3 and 4 show that the IGHD3 paralog does not exist (e.g. was deleted) in cluster 2.

**Key**

Green – Family 1

Light Green – Family 2

Red – Family 3

Purple – Family 7

Blue – IGHD8-2

Gold – Family 5

Orange – Family 6

Lowercase – RSS, with heptamer and nonamer in italics and underlined

#NEXUS

begin data;

dimensions ntax=3 nchar=3954;

format datatype=dna interleave=yes gap=-;

matrix

'Dcluster2' ***ggattttga***gggtgtgcgtgt***caccctg***AGAATATCGTGATGATGGTTACTGCTACACC***c***

'Dcluster3' ***ggattttga***gggtgtgcgtgt***caccgtg***AGACTATCGTGATGATGGTTACTGCTACACC***c***

'Dcluster4' ***ggattttga***gggtgtgcgtgt***caccgtg***AGAATATCGTGATGATGGTTACTGCTACACC***c***

'Dcluster2' ***acagtg***actcaggccctg***acataaagt***CTGACCCGCACACAGGTGTGGAGCTTGCCAATG

'Dcluster3' ***acagtg***actcaggccctg***acataaagt***CTGACCCGCACACAGGTGTGGAGCTGGCCAATG

'Dcluster4' ***acagtg***actcaggccctg***acataaagt***CTGACCCGCACACAGGTGTGGAGCTGGCCAATG

'Dcluster2' CATCCCCAGGGGCACTGGGCTCCCAAGCAAGGTGCCTATCCCCCCAACTCGGGACAGTGA

'Dcluster3' CATCCCCAGGGGCACTGGGCTCCCAAGCAAGGTGCCTATCCCCCCAACTCGGGACAGTGA

'Dcluster4' CATCCCCAGGGGCACTGGGCTCCCAAGCAAGGTGCCTATCCCCCCAACTCGGGACAGTGA

'Dcluster2' GCCAGAAGGGATGTGTGGGCGTGGACCCTGGGCTCAGCCCACGGCCAGAGGACAGGGAAG

'Dcluster3' GCCAGAAGGGATGTGTGGGCGTGGACCCTGGGCTCAGCCCACGGCCAGAGGACAGGGAAG

'Dcluster4' GCCAGAAGGGATGTGTGGGCGTGGACCCTGGGCTCAGCCCACGGCCAGAGGACAGGGAAG

'Dcluster2' GGCCCAGGGCTCCAGGCCACCTACCGAGAGCATTGAGCAAAGGTGGCTGAGGCCCCAATG

'Dcluster3' GGCCCAGGGCTCCAGGCCACCTACCGAGAGCATTGAGCAAAGGTAGCTGAGGCCCCAATG

'Dcluster4' GGCCCAGGGCTCCAGGCCACCTACCGAGAGCATTGAGCAAAGGTAGCTGAGGCCCCAATG

'Dcluster2' AGGCAGCCATCCCAGGGAGCCCCTGACCCCTCCCCAGGACCAAGACTTGCCAGCAGGGCA

'Dcluster3' AGGCAGCCATCCCAGGGAACCCCTGACCCCTCCCCAGGACCAAGACTTGCCAGCAGGGCA

'Dcluster4' AGGCAGCCATCCCAGGGAACCCCTGACCCCTCCCCAGGACCAAGACTTGCCAGCAGGGCA

'Dcluster2' GGGGTCCCGGCAAAGGGCCAGCCCTCCAGCCCTGGTGCACGCAC-CCCCTGCAGGCGAAG

'Dcluster3' GGGGTCCCGGCAAAGGGCCAGCCCTCCAGCCCTGGTGCACACACCCCCCTGCAGGCGAAG

'Dcluster4' GGGGTCCCGGCAAAGGGCCAGCCCTCCAGCCCTGGTGCACACACCCCCCTGCAGGCGAAG

'Dcluster2' GGGGCACCTCTCGATTCCCCGGGGTGCTAGGGGGAGACAAGATCCAGGGGACTGTCCACA

'Dcluster3' GGGGCACCTCTCGATTCCCCGGGGTGCTAGGGGGAGACAAGATCCAGGGGACTGTCCACA

'Dcluster4' GGGGCACCTCTCGATTCCCCGGGGTGCTAGGGGGAGACAAGATCCAGGGGACTGTCCACA

'Dcluster2' TTCCAGCAGGGACTCTGCTGGACAAGCCAGGGCTTCCAGGCCTGATCACAATCCATCACC

'Dcluster3' TTCCAGCAGGGACTCTGCTGGACAAGCCAGGGCTTCCAGGCCTGATCACAACCCATCACC

'Dcluster4' TTCCAGCAGGGACTCTGCTGGACAAGCCAGGGCTTCCAGGCCTGATCACAACCCATCACC

'Dcluster2' CAAAATCAGCATGG-----GTGCCCAGGGGCCACTGAGGGCCTCAAGAAGGTGGGTCTCA

'Dcluster3' CAAAATCAGCGTGGAGTGGGTGCCCAGGGGCCACTGAGGGCCTCAAGAAGGTGGGTCTCA

'Dcluster4' CAAAATCAGCGTGGAGTGGGTGCCCAGGGGCCACTGAGGGCCTCAAGAAGGTGGGTCTCA

'Dcluster2' ACCGTGCTCCATGGCCTGGAGCCGTGAGGGCACAGAGGGCATGAACCGACTGCAGAGGAG

'Dcluster3' ACCGTGCTCCATGGCCTGGAGCCGTGAGGGCACAGAGGGCATGAACCGACTGCGGAGGAG

'Dcluster4' ACCGTGCTCCATGGCCTGGAGCCGTGAGGGCACAGAGGGCATGAACCGACTGCGGAGGAG

'Dcluster2' GGCCCTGCCCCTCAGCGAATGCCTCGTCCTGGGCCTCTGGGCCCCCGGGGTCAGGGCGGG

'Dcluster3' GGCCCTGCCCCTCAGCGAATGCCTCGTCCTGGGCCTCTGGGCCCCCGGGGTCAGGGCGGG

'Dcluster4' GGCCCTGCCCCTCAGCGAATGCCTCGTCCTGGGCCTCTGGGCCCCCGGGGTCAGGGCGGG

'Dcluster2' GACGGGTGCAGGGTAGGGGGATGCAGCTCCCTGCACCTGCTCCCAGTCCCGCTGGGGCCA

'Dcluster3' GACGGGTGCAGGGTAGGGGGATGCAGCTCCCTGCACCTGCTCCCAGTCCTGCTGGGGCCA

'Dcluster4' GACGGGTGCAGGGTAGGGGGATGCAGCTCCCTGCACCTGCTCCCAGTCCTGCTGGGGCCA

'Dcluster2' CGAGGATGAGCAGCTCAGCACTGCCCTCTGGGCTGGGCCAGCACAACTGGGCCCAGACTC

'Dcluster3' CAAGGATGAGCAGCTCAGCACTGCCCTGTGGGCTGGGCCAGCACAACTGGGCCCAGACTC

'Dcluster4' CAAGGATGAGCAGCTCAGCACTGCCCTGTGGGCTGGGCCAGCACAACTGGGCCCAGACTC

'Dcluster2' CCAGGACGGGCAGCAGCAGGAATGACCCTGCTCTGCTCCTGGGTTTCCTGGCGGGAGGCG

'Dcluster3' CCAGGACGGGCAGCAGCAGGAATGACCCTGCTCTGCTCCTGGGTTTCCTGGCGGGAGGCG

'Dcluster4' CCAGGACGGGCAGCAGCAGGAATGACCCTGCTCTGCTCCTGGGTTTCCTGGCGGGAGGCG

'Dcluster2' CCTACTGACCCCAGAGCACAACTGGCCTCAAGGTGACCGATACACAGTTTGTGGGCAATT

'Dcluster3' CCTGCTGGCCCCAGAGCACAACCGGCCTCAAGGTGACCGATACACAGTTTGCAGGCAA-C

'Dcluster4' CCTGCTGGCCCCAGAGCACAACCGGCCTCAAGGTGACCGATACACAGTTTGCAGGCAA-C

'Dcluster2' TGGGTGCAATCTGTCAACCCAGGAGGCTGTCACTTGGCCAAGCCCAGCTTGGGGCCTCAG

'Dcluster3' TGGGTAAAATCTGTCAACCCAGGAGGCTGTCACTTGGCTGAGCCCAGCTCGGGGCCTCAG

'Dcluster4' TGGGTAAAATCTGTCAACCCAGGAGGCTGTCACTTGGCTGAGCCCAGCTCGGGGCCTCAG

'Dcluster2' GAGAGCCAGCCTGGTCATCGTCCACCTGGCAGGAGACGCCTCCCACCGGCTCCAGCAGGT

'Dcluster3' GAGAGCCAGCCTGGTCATCGTCCACCTGGCAGGAGATGCCTCCCAGGTGCTCCAGCAGGT

'Dcluster4' GAGAGCCAGCCTGGTCATCGTCCACCTGGCAGGAGATGCCTCCCAGGTGCTCCAGCAGGT

'Dcluster2' CGCCTGTGGCACCATGCCCTCCACAAGCCCTGAGCCCAGCACCAACCCACCCACAGCA--

'Dcluster3' CTCCTGTGGCGCCATGCCCTCCACAAGCCCTGAGCCTGGCACCAACCCACCCACAGCCAG

'Dcluster4' CTCCTGTGGCGCCATGCCCTCCACAAGCCCTGAGCCTGGCACCAACCCACCCACAGCCAG

'Dcluster2' --CAGGCTGCCCAGCTCCCATGACTGTTCAGCCCTCTGAACGCCCCACATCCAGGGTCCC

'Dcluster3' CCCAGGCCGCCCAGCCCCCATGACTGTTCAGCCCTCTGAACGCCCCACATCCAGGGTCCC

'Dcluster4' CCCAGGCCGCCCAGCCCCCATGACTGTTCAGCCCTCTGAACGCCCCACATCCAGGGTCCC

'Dcluster2' CACGCAGACGGCCTACAGCACGAACGGG***gctttttgc***caagggctctac***tgcggtg***TTAC

'Dcluster3' CACGCAGACGGCCTACAGCGCAAACGGG***gctttttgc***caagggctctac***tgcggtg***TTAC

'Dcluster4' CACGCAGACGGCCTACAGTGCAAACGGG***gctttttgc***caagggctctac***tgcggtg***TTAC

'Dcluster2' TATAGTGACCAC***cacagtg***agacatggggca***gcaaaccct***GCCAACCGTATGGCCAACCA

'Dcluster3' TATAGTGACCAC***cacagtg***agacatggggca***gcaaaccct***GCCAACCGTATGGCCAACCA

'Dcluster4' TATAGTGACCAC***cacagtg***agacatggggca***gcaaaccct***GCCAACCGTATGGCCACCCA

'Dcluster2' GCCCCCACGGCCGGGCCTCACGGCCTGAGCTTCAGAGACCAGACCTTCGGCCTTCTGGGC

'Dcluster3' GCCCCCACGGCCGGGCCTCACGGCCTGAGCTTCAGAGACCAGACCTTCGGCCATCTGGGC

'Dcluster4' GCCCCCACGGCCGGGCCTCACGGCCTGAGCTTCAGAGACCAGACCTTCGGCCATCTGGGC

'Dcluster2' CACTCTGGGCGGATGTGGCCCGCGGGCCCGTATCCAGACTCTGAGATGACTGCACTGACC

'Dcluster3' CACTCTGGGCGGATGCGGCCCACGGGCCCATATCCAGACTCTGAGATGACTGCACTGACC

'Dcluster4' CACTCTGGGCGGATGCGGCCCATGGGCCCATATCCAGACTCTGAGATGACTGCACTGACC

'Dcluster2' CTCCAGCCACACGTCCTAGCCCAGCAAGCTGGCCCTGACCACTAGCACTCAGTACACCCC

'Dcluster3' CTCCAGCCACACATCCTAGCCCAGCAAGCTGGCCCTGACCACTAGCACTCAGTACACCCC

'Dcluster4' CTCCAGCCACACATCCTAGCCCAGCAAGCTGGCCCTGACCACTAGCACTCAGTACACCCC

'Dcluster2' CAAACAGGGTCAGGAAGGACAGAGCCCACGCTGGGCAAACCGTCAGGTCAAGTTTCCCAG

'Dcluster3' CAAACAGGGTCAGGAAGGACAGAGCCCACGCTGGGCAAACCGTCAGGTCAAGTTTCCCAG

'Dcluster4' CAAACAGGGTCAGGAAGGACAGAGCCCACGCTGGGCAAACCGTCAGGTCAAGTTTCCCAG

'Dcluster2' GGGCAGGACCCGAGACACCTCCAACCCTGCTGGGAGCTCAGGGAAGCTCACAGCAGTGGA

'Dcluster3' GGGCAGGACCTGAGACACCTCCAACCCTGCTGGGAGCTCAGGGAAGCTCACAGCAGTGGA

'Dcluster4' GGGCAGGACCCGAGACACCTCCAACCCTGCTGGGAGCTCAGGGAAGCTCACAGCAGTGGA

'Dcluster2' CGCTGGAGCTGGGCCTGTACTCAACTGCATAGGAGCTCACCAGGGAGCAGGCTGGGAGGG

'Dcluster3' CGCTGGAGCTGGGCC-GTACTCAACTGCATAGGAGCTCGCCAGGGAGCAGGCTGGGAGGG

'Dcluster4' TGCTGGAGCTGGGCCTGTACTCAACTGCATAGGAGCTCGCCAGGGAGCAGGCTGGGAGGG

'Dcluster2' GTTCATGCAGAGGGAGCAGCTCCCAGCATGCTGCCAGACCCGAGGTAGGGGAGTCCGGAT

'Dcluster3' GTTCATGCAGAGGCAGCAGCTCCCAGCATGCTGCCAGACCCGAGGTAGGGGAGTCCGGAT

'Dcluster4' GTTCATGCAGAGGCAGCAGCTCCCAGCATGCTGCCAGACCCGAGGTAGGGGAGTCCGGAT

'Dcluster2' CCAGGCCCGGCCTGAAGGGGCTCGGGGGGCCTCTCACATGTAGCAGGGGTGTGCAGGTCC

'Dcluster3' CCAGGCCCGGCCTGAAGGGGCTCGGGGGGCCTCTCACATGTAGCAGGGGTGTGCAGGTCC

'Dcluster4' CCAGGCCCGGCCTGAAGGGGCTCGAGGGGCCTCTCACATGTAGCAGGGGTGTGCAGGTCC

'Dcluster2' CTTGTGCAGTGTCCAGAGATCATGGACAGGGCGGGCACCGCACCAGAAGCCAAGCCAGTG

'Dcluster3' CTTGTGCAGTGTCCAGAGATCATGGACAGGGCGGGCACTGCACCAGAAGCCAAGCCAGTG

'Dcluster4' CTTGTGCAGTGTCCAGAGATCATGGACAGGGCGGGCACTGCACCAGAAGCCAAGCCAGTG

'Dcluster2' ACCAGCCTCTGCAGGAAGCACCCATGGGAGCCAACACCTAAGTCTCCCTCCCAGCCCTGC

'Dcluster3' ACCAGCCTCTGCAGGAAGCGCCCGTGGGAGCCAACACCTAAGTCTCCCTCCCAGCCCTGC

'Dcluster4' ACCAGCCTCTGCAGGAAGCGCCCGTGGGAGCCAACACCTAAGTCTCCCTCCCAGCCCTGC

'Dcluster2' AGCGTGGCCACCCACCACCCCACAGGTCAGCCTGGGGAGCTCAAGACGGACTGGCGTCCC

'Dcluster3' AGCGTGGCCACCCACCACCCCACAGGTCAGCCTGGGGAGCTCAAGACGGACTGGCGTCCC

'Dcluster4' AGCGTGGCCACCCACCACCCCACAGGTCAGCCTGGGGAGCTCAAGACGGACTGGCGTCCC

'Dcluster2' TACCTGCCAGGTTCCTGTGCAGGATGCTGTCCACGGCCGCAATGCTTTGGCCTCTGCTCA

'Dcluster3' TACCTGCCAGGTTCCTGTGCAGGACGCTGTCCACAGGCGCAATGCTTGGGCCTCTGCTCA

'Dcluster4' TACCTGCCAGGTTCCTGTGCAGGACGCTGTCCACAGGCGCAATGCTTGGGCCTCTGCTCA

'Dcluster2' GCTTCTGAGGTTCTCCGGCCCACCACCACTGACCTGAATGGGCTTGGAGTGCAGGCCCAG

'Dcluster3' GCTTCCGAGGTTCCCTGGCCCACCACCACCGACCTGAAGGGGCTCGGAGTGCAGGCCCAG

'Dcluster4' GCTTCCGAGGTTCCCTGGCCCACCACCACCGACCTGAAGGGGCTCGGAGTGCAGGCCCAG

'Dcluster2' CTGAACATCAGACCTGGGCTCAGAGAGCCACTCGGTCCAGGGTGCACTCAGCCGTGCACC

'Dcluster3' CAGGACATAAGACCTGGGCTCAGATAGCCACTCGGTCCAGGGTGCACTCAGCCCTGCACC

'Dcluster4' CAGGACATAAGACCTGGGCTCAGATAGCCACTCGGTCCAGGGTGCACTCAGCCCTGCACC

'Dcluster2' TGGAGGAGGGTCCTCCATG-CCGCAGGGTTGGCTGGGCAAGGAGGCATCACAGACCACCC

'Dcluster3' TGGAGGTGGGTCCTCCATGGCCGCAGCATTGGCTGGGCAAGGAGGCATCCCAGGCCACCC

'Dcluster4' TGGAGGTGGGTCCTCCATGGCCGCAGCATTGGCTGGGCAAGGAGGCATCCCAGGCCACCC

'Dcluster2' TGCTGTTGGGACGTCCAGCCCTGGACCAGGACTAGGTTCTCAGAATCCTCCCCACCACGG

'Dcluster3' TGCCGTTGGGCCGTCCAGCCCTGGACCAGGACTAGGTTCTCAGAACCCTCCCCACCACGG

'Dcluster4' TGCCGTTGGGCCGTCCAGCCCTGGACCAGGACTAGGTTCTCAGAACCCTCCCCACCACGG

'Dcluster2' GACCTGTCCATACCTGGACCAGCTGACAGCTCCCAAGGGCTCCA-ACCCAGCAGACAAGA

'Dcluster3' GACCCGTCCAGACCTGGGCCAGCTGACAGCTCCCAAGGGCTCCACACCCAGCAGACAAGA

'Dcluster4' GACCCGTCCAGACCTGGGCCAGCTGACAGCTCCCAAGGGCTCCACACCCAGCAGACAAGA

'Dcluster2' CCAA--GGGCGCTGAAGGCGAGCAGCTAGG***gtttctga***tgccggctgtgt***tgtggtg***ATG

'Dcluster3' CCAAGGGGGCGCTGAAAACCAACAACTGGG***gtttctga***tgccggctgtgt***cacggtg***GTA

'Dcluster4' CCAAGGGGGCGCTGAAAACCAACAACTGGG***gtttctga***tgccggctgtgt***cacggtg***GTA

'Dcluster2' ATACGATAGGTGTGGTTGTAGTTATTGTAGTGTTGCTAC***cacagtg***atgctctcagtg***tc***

'Dcluster3' TTGTGGTAGCTATTGTGGTAGTTATTATGGT------AC***cacagtg***acactgtccagg***ac***

'Dcluster4' TTGTGGTAGCTATTGTGGTAGTTATTATGGT------AC***cacagtg***acactgtccagg***ac***

'Dcluster2' ***agaaacc***CCTGCCCCGAGGTCCCAGACCAGGGAGCCTGTCCTCCAGGTACGGGGCACCGC

'Dcluster3' ***agaaacc***CCCGTCCCAAGGTCCCAGACCAGGGAGCCTGTCTTCCAAGACCGGGGTACCGC

'Dcluster4' ***agaaacc***CCCGTCCCAAGGTCCCAGACCAGGGAGCCTGTCTTCCAAGACCGGGGCACCGC

'Dcluster2' CCTGCCCTCGTGACCATCAGACTGCACCCTGCACGCCCTCACCCTGCTGTCTCAGGGCAG

'Dcluster3' CCTGCCCTCCTGACCATCAGACTGCACCCTGCACGCCCTCACCCCACTGGCTCAGGGAAG

'Dcluster4' CCTGCCCTCCTGACCATCAGACTGCACCCTGCACGCCCTCACCCCACTGGCTCAGGGAAG

'Dcluster2' CAACAGCCTCTGGAGGAGTCCTGGGCCAGGCACTCGGCTGTGGAGTCCCAGACAATGATA

'Dcluster3' CAACAGCCTGAGGAAGGGTCCTGGGCCAGGCGCTGGGCTATGGGGACCCAGACAATGAGA

'Dcluster4' CAACAGCCTGAGGAAGGGTCCTGGGCCAGGCGCTGGGCTATGGGGACCCAGACAATGAGA

'Dcluster2' GCCTCAGCAGTTTCCAGCCACCTGACCCTGGAGGAGCTTGTGCAAGCTGGAGGCC-----

'Dcluster3' CCCCCAGCAGGTTCCAGCCCCCTGACCCTGGAGGAACTT-------CCAGAGGCCTGTGC

'Dcluster4' CCCCCAGCAGGTTCCAGCCCCCTGACCCTGGAGGAACTT-------CCAGAGGCCTGTGC

'Dcluster2' --GGCTGGGGCCCGGAGCCTCAGAACCCTCCCTGCCAGGGGGCCCGTCCAGACCTGGACC

'Dcluster3' CAGGCTGGGGACC-GACCCTAAGAACCCTCCCC-CGACAGGGCCCATCCAGA--------

'Dcluster4' CAGGCTGGGGACC-GACCCTAAGAACCCTCCCC-CGACAGGGCCCATCCAGA--------

'Dcluster2' AGCTGACAGCTCCCGAGGACTGCACGCCCACCAGACGAGACCAAGGGGGCGCTGAAGGCC

'Dcluster3' ------CAGCTCTCGAGGTCTCCATGTCCACCAGACAAGACCAAGGGGGCGCTGAAGGCC

'Dcluster4' ------CAGCTCTCGAGGTCTCCATGTCCACCAGACAAGACCAAGGGGGCGCTGAAGGCC

'Dcluster2' AGCAGCTGG***ggtttctga*tgccggctgtgt*cacggtg*GTAGTTGTCCTGATGGTTATAGT**

'Dcluster3' AGCAACTGG***ggtttttga***tgccggctgtgt***cacggtg***GTAGTT------ATGGT------

'Dcluster4' AGCAACTGG***ggtttttga***tgccggctgtgt***cacggtg***GTAGTT------ATGGT------

'Dcluster2' **TATGGTTATGGTTGTGGTTATGGTTATGGTTGTAGTGGTTATGATTGTTATGGTTATGGT**

'Dcluster3' ---------------------GGTTATGGTTATGGTGGTTATGGTTGTT------ATGGT

'Dcluster4' ---------------------GGTTATGGTTATGGTGGTTATGGTTGTTATGGTTATGGT

'Dcluster2' **GGTTATGGTGGTTATGGTGGTTATGGTTATAGTAGTTATAGTTATAGTTATACTTACGAA**

'Dcluster3' ---TATGGT------------TATGGTTATGGT---------------------------

'Dcluster4' ---TATGGT------------TATGGTTATGGT---------------------------

'Dcluster2' **TATAC*cacagtg*atactctctggg*acaaaaacc***CCTGCCCCTGAGGGTCCACGGCCAGGG

'Dcluster3' TATAC***cacagtg***atactctctggg***acaaaaacc***CCTGCCCCTGAGGGTCCACGACCAGGG

'Dcluster4' TATAC***cacagtg***atactctctggg***acaaaaacc***CCTGCCCCTGAGGGTCCACGACCAGGG

'Dcluster2' ATCCTGGAGGCTGTGTTTGGGGCACCGCCCTGCCCTCCTGCCATTGGACTACAACCTGCA

'Dcluster3' AATCTGTAAGCTGTGTTTGGGGCACCACCCTGCCCTCCTGCCATTGGACTGCACCCTGCA

'Dcluster4' AATCTGTAAGCTGTGTTTGGGGCACCACCCTGCCCTCCTGCCATTGGACTGCACCCTGCA

'Dcluster2' CGCCCTCACCCTGCTGACTCGGGCAGCAACAGCCTAAGATAAGAGTCCTGGGCCAGGCGC

'Dcluster3' CGCCCTCACCCTGCTGACTCGGGCAGCAACAGCCTAAGATAAGGGTCCTGGGCCAGGAGC

'Dcluster4' CGCCCTCACCCTGCTGACTCGGGCAGCAACAGCCTAAGATAAGGGTCCTGGGCCAGGAGC

'Dcluster2' TGGGCTGTGGATACCCAGACAATGAGATCTCCAGCAGGTTCCCACCTCCTGACCCTGGAG

'Dcluster3' TGGGCTGTGGATACCCAGACAATGAGATCTCCAGCAGGTTCCCGCCTCCTGATC-TGGAG

'Dcluster4' TGGGCTGTGGATACCCAGACAATGAGATCTCCAGCAGGTTCCCGCCTCCTGATC-TGGAG

'Dcluster2' GAACTTCCGCAGCCCTGTGGCAGGCTGGGGACCGACCCTAAGAACCCT-CCCCCCGC---

'Dcluster3' GAACTGCCGCAGGCCTGTGCCAGGCTGGGGACCGACCCTAAGAACCCTCCCCCCCGCAGG

'Dcluster4' GAACTGCCGCAGGCCTGTGCCAGGCTGGGAACCGACCCTAAGAACCCT-CCCCCCGCAGG

'Dcluster2' ------------------------------------------------------------

'Dcluster3' GCCATCCAGACCTGGGCCAGCTGACAGCTCCCGAGGACTCCACGTCCACCAGACGAGACC

'Dcluster4' GCCATCCAGACCTGGGCCAGCTGACAGCTCCCGAGGACTCCACGTCCACCAGACGAGACC

'Dcluster2' ------------------------------------------------------------

'Dcluster3' AAGGGGACGCCGAAGGCCAGCAGCTGG***ggtttctga***tgccggctgtgt***tgtggtg***ATGAT

'Dcluster4' AAGGGGACGCCGAAGGCCAGCAGCTGG***ggtttctga***tgccggctgtgt***tgtggtg***ATGAT

'Dcluster2' ------------------------------------------------------------

'Dcluster3' ACGATAGGTGTGGTTTTAGTTATTGTAGTGTTGCTAC***cacagtg***acgctctcagtg***tcag***

'Dcluster4' ACGATAGGTGTGGTTTTAGTTATTGTAGTGTTGCTAC***cacagtg***acgctctcagtg***tcag***

'Dcluster2' ------------------------------------------------------------

'Dcluster3' ***aaacc***CCTGCCCCGAGGTCCCAGACCAGGGAGCTTGTCCTCCAGGTCCGGGGTACGGCCC

'Dcluster4' ***aaacc***CCTGCCCCGAGGTCCCAGACCAGGGAGCTTGTCCTCCAGGTCCGGGGTATGGCCC

'Dcluster2' ------------------------------------------------------------

'Dcluster3' TGCCCTCCTGACCATCGGACTGCACCCTGCATGCCCTCACCCTGCTGACTCAGGGCAGCA

'Dcluster4' TGCCCTCCTGACCATCGGACTGCACCCTGCATGCCCTCACCCTGCTGACTCAGGGCAGCA

'Dcluster2' ------------------------------------------------------------

'Dcluster3' ACAGAATGAGGAAGGGTCCTGGGCCAAGAGCTTGGCTGCTGGGACCCAGACAATGAGGCC

'Dcluster4' ACAGAATGAGGAAGGGTCCTGGGCCAAGAGCTTGGCTGCTGGGACCCAGACAATGAGGCC

'Dcluster2' ------------------------------------------------------------

'Dcluster3' CCCAGCAAGTTCCAGCTCCCTGACCCTGGAGGAGGTTGTGCAGGTCAGAGGCCGGCTGGG

'Dcluster4' CCCAGCAAGTTCCAGCCCCCTGACCCTGGAGGAGGTTGTGCAGGTCAGAGGCCGGCTGGG

'Dcluster2' -----------------------------AGGGCCCGTCCAGACCTGAACCAGCTGACAG

'Dcluster3' GACCGGATCCTCAGAACCCTCCCTGCCGCAGGGCCCGTCCAGA-CTGGACCAGCTGACAG

'Dcluster4' GACCGGATCCTCAGAACCCTCCCTGCCGCAGGGCCCGTCCAGA-CTGGACCAGCTGACAG

'Dcluster2' CTCCCGAGGGCTCCACGCCCCCCAGACGAGACCAAGGGGGCGCCGAAGGCCAGCAGCTGG

'Dcluster3' CTCCCAAGGACTCCACGCCCACCAGACGAGACCAAGGGGGCGCCGAAGGCCAGCAGCCGG

'Dcluster4' CTCCCAAGGACTCCACGCCCACCAGACGAGACCAAGGGGGCGCCGAAGGCCAGCAGCCGG

'Dcluster2' ***ggtttctga***tgccggctgtgt***cacggtg***GTAGTTGTTATAGTGGTTATGGTTATGGTTGT

'Dcluster3' ***ggtttctga***tgccagctgtgt***cacggtg***GTAGTTGTTATAGTGGTTATGGTTATGGTTAT

'Dcluster4' ***ggtttctga***tgccagctgtgt***cacggtg***GTAGTTGTTATAGTGGTTATGGTTATGGTTAT

'Dcluster2' GGTTATGGTTATGGTTATGATTATAC***cacagtg***acactctctggg***acaaaaacc***

'Dcluster3' GGTTGTGGTTATGGTTATGGTTATAC***cacagtg****a*cactctctggg***acaaaaacc***

'Dcluster4' GGTTGTGGTTATGGTTATGGTTATAC***cacagtg***acactctctggg***acaaaaacc***

;

end;

########################################

# Program: stretcher

# Rundate: Sat 17 Aug 2019 01:07:27

# Commandline: stretcher

# -auto

# -stdout

# -asequence emboss_stretcher-I20190817-010723-0557-75507456-p2m.asequence

# -bsequence emboss_stretcher-I20190817-010723-0557-75507456-p2m.bsequence

# -datafile EDNAFULL

# -gapopen 25

# -gapextend 1

# -aformat3 pair

# -snucleotide1

# -snucleotide2

# Align_format: pair

# Report_file: stdout

########################################

#=======================================

#

# Aligned_sequences: 2

# 1: Dcluster2

# 2: Dcluster3

# Matrix: EDNAFULL

# Gap_penalty: 25

# Extend_penalty: 1

#

# Length: 3947

# Identity: 3185/3947 (80.7%)

# Similarity: 3185/3947 (80.7%)

# Gaps: 574/3947 (14.5%)

# Score: 14191

#

#

#=======================================

Dcluster2 1 ***ggattttga***gggtgtgcgtgt***caccctg***AGAATATCGTGATGATGGTTAC 50

|||||||||||||||||||||||||.|||||.||||||||||||||||||

Dcluster3 1 ***ggattttga***gggtgtgcgtgt***caccgtg***AGACTATCGTGATGATGGTTAC 50

Dcluster2 51 TGCTACACC***cacagtg***actcaggccctg***acataaagt***CTGACCCGCACAC 100

||||||||||||||||||||||||||||||||||||||||||||||||||

Dcluster3 51 TGCTACACC***cacagtg***actcaggccctg***acataaagt***CTGACCCGCACAC 100

Dcluster2 101 AGGTGTGGAGCTTGCCAATGCATCCCCAGGGGCACTGGGCTCCCAAGCAA 150

||||||||||||.|||||||||||||||||||||||||||||||||||||

Dcluster3 101 AGGTGTGGAGCTGGCCAATGCATCCCCAGGGGCACTGGGCTCCCAAGCAA 150

Dcluster2 151 GGTGCCTATCCCCCCAACTCGGGACAGTGAGCCAGAAGGGATGTGTGGGC 200

||||||||||||||||||||||||||||||||||||||||||||||||||

Dcluster3 151 GGTGCCTATCCCCCCAACTCGGGACAGTGAGCCAGAAGGGATGTGTGGGC 200

Dcluster2 201 GTGGACCCTGGGCTCAGCCCACGGCCAGAGGACAGGGAAGGGCCCAGGGC 250

||||||||||||||||||||||||||||||||||||||||||||||||||

Dcluster3 201 GTGGACCCTGGGCTCAGCCCACGGCCAGAGGACAGGGAAGGGCCCAGGGC 250

Dcluster2 251 TCCAGGCCACCTACCGAGAGCATTGAGCAAAGGTGGCTGAGGCCCCAATG 300

||||||||||||||||||||||||||||||||||.|||||||||||||||

Dcluster3 251 TCCAGGCCACCTACCGAGAGCATTGAGCAAAGGTAGCTGAGGCCCCAATG 300

Dcluster2 301 AGGCAGCCATCCCAGGGAGCCCCTGACCCCTCCCCAGGACCAAGACTTGC 350

||||||||||||||||||.|||||||||||||||||||||||||||||||

Dcluster3 301 AGGCAGCCATCCCAGGGAACCCCTGACCCCTCCCCAGGACCAAGACTTGC 350

Dcluster2 351 CAGCAGGGCAGGGGTCCCGGCAAAGGGCCAGCCCTCCAGCCCTGGTGCAC 400

||||||||||||||||||||||||||||||||||||||||||||||||||

Dcluster3 351 CAGCAGGGCAGGGGTCCCGGCAAAGGGCCAGCCCTCCAGCCCTGGTGCAC 400

Dcluster2 401 GCACCCCC-TGCAGGCGAAGGGGGCACCTCTCGATTCCCCGGGGTGCTAG 449

.||||||| |||||||||||||||||||||||||||||||||||||||||

Dcluster3 401 ACACCCCCCTGCAGGCGAAGGGGGCACCTCTCGATTCCCCGGGGTGCTAG 450

Dcluster2 450 GGGGAGACAAGATCCAGGGGACTGTCCACATTCCAGCAGGGACTCTGCTG 499

||||||||||||||||||||||||||||||||||||||||||||||||||

Dcluster3 451 GGGGAGACAAGATCCAGGGGACTGTCCACATTCCAGCAGGGACTCTGCTG 500

Dcluster2 500 GACAAGCCAGGGCTTCCAGGCCTGATCACAATCCATCACCCAAAATCAGC 549

|||||||||||||||||||||||||||||||.||||||||||||||||||

Dcluster3 501 GACAAGCCAGGGCTTCCAGGCCTGATCACAACCCATCACCCAAAATCAGC 550

Dcluster2 550 ATGG-----GTGCCCAGGGGCCACTGAGGGCCTCAAGAAGGTGGGTCTCA 594

.||| |||||||||||||||||||||||||||||||||||||||||

Dcluster3 551 GTGGAGTGGGTGCCCAGGGGCCACTGAGGGCCTCAAGAAGGTGGGTCTCA 600

Dcluster2 595 ACCGTGCTCCATGGCCTGGAGCCGTGAGGGCACAGAGGGCATGAACCGAC 644

||||||||||||||||||||||||||||||||||||||||||||||||||

Dcluster3 601 ACCGTGCTCCATGGCCTGGAGCCGTGAGGGCACAGAGGGCATGAACCGAC 650

Dcluster2 645 TGCAGAGGAGGGCCCTGCCCCTCAGCGAATGCCTCGTCCTGGGCCTCTGG 694

|||.||||||||||||||||||||||||||||||||||||||||||||||

Dcluster3 651 TGCGGAGGAGGGCCCTGCCCCTCAGCGAATGCCTCGTCCTGGGCCTCTGG 700

Dcluster2 695 GCCCCCGGGGTCAGGGCGGGGACGGGTGCAGGGTAGGGGGATGCAGCTCC 744

||||||||||||||||||||||||||||||||||||||||||||||||||

Dcluster3 701 GCCCCCGGGGTCAGGGCGGGGACGGGTGCAGGGTAGGGGGATGCAGCTCC 750

Dcluster2 745 CTGCACCTGCTCCCAGTCCCGCTGGGGCCACGAGGATGAGCAGCTCAGCA 794

|||||||||||||||||||.|||||||||||.||||||||||||||||||

Dcluster3 751 CTGCACCTGCTCCCAGTCCTGCTGGGGCCACAAGGATGAGCAGCTCAGCA 800

Dcluster2 795 CTGCCCTCTGGGCTGGGCCAGCACAACTGGGCCCAGACTCCCAGGACGGG 844

|||||||.||||||||||||||||||||||||||||||||||||||||||

Dcluster3 801 CTGCCCTGTGGGCTGGGCCAGCACAACTGGGCCCAGACTCCCAGGACGGG 850

Dcluster2 845 CAGCAGCAGGAATGACCCTGCTCTGCTCCTGGGTTTCCTGGCGGGAGGCG 894

||||||||||||||||||||||||||||||||||||||||||||||||||

Dcluster3 851 CAGCAGCAGGAATGACCCTGCTCTGCTCCTGGGTTTCCTGGCGGGAGGCG 900

Dcluster2 895 CCTACTGACCCCAGAGCACAACTGGCCTCAAGGTGACCGATACACAGTTT 944

|||.|||.||||||||||||||.|||||||||||||||||||||||||||

Dcluster3 901 CCTGCTGGCCCCAGAGCACAACCGGCCTCAAGGTGACCGATACACAGTTT 950

Dcluster2 945 GTGGGCAATTTGGGTGCAATCTGTCAACCCAGGAGGCTGTCACTTGGCCA 994

|..|||||.| ||||..|||||||||||||||||||||||||||||||..

Dcluster3 951 GCAGGCAACT-GGGTAAAATCTGTCAACCCAGGAGGCTGTCACTTGGCTG 999

Dcluster2 995 AGCCCAGCTTGGGGCCTCAGGAGAGCCAGCCTGGTCATCGTCCACCTGGC 1044

|||||||||.||||||||||||||||||||||||||||||||||||||||

Dcluster3 1000 AGCCCAGCTCGGGGCCTCAGGAGAGCCAGCCTGGTCATCGTCCACCTGGC 1049

Dcluster2 1045 AGGAGACGCCTCCCACCGGCTCCAGCAGGTCGCCTGTGGCACCATGCCCT 1094

||||||.||||||||...|||||||||||||.||||||||.|||||||||

Dcluster3 1050 AGGAGATGCCTCCCAGGTGCTCCAGCAGGTCTCCTGTGGCGCCATGCCCT 1099

Dcluster2 1095 CCACAAGCCCTGAGCCCAGCACCAACCCACCCACAGCA----CAGGCTGC 1140

||||||||||||||||..|||||||||||||||||||. |||||.||

Dcluster3 1100 CCACAAGCCCTGAGCCTGGCACCAACCCACCCACAGCCAGCCCAGGCCGC 1149

Dcluster2 1141 CCAGCTCCCATGACTGTTCAGCCCTCTGAACGCCCCACATCCAGGGTCCC 1190

|||||.||||||||||||||||||||||||||||||||||||||||||||

Dcluster3 1150 CCAGCCCCCATGACTGTTCAGCCCTCTGAACGCCCCACATCCAGGGTCCC 1199

Dcluster2 1191 CACGCAGACGGCCTACAGCACGAACGGG***gctttttgc***caagggctctac***t*** 1240

|||||||||||||||||||.|.||||||||||||||||||||||||||||

Dcluster3 1200 CACGCAGACGGCCTACAGCGCAAACGGG***gctttttgc***caagggctctac***t*** 1249

Dcluster2 1241 ***gcggtg***TTACTATAGTGACCAC***cacagtg***agacatggggca***gcaaaccct*** 1290

||||||||||||||||||||||||||||||||||||||||||||||||||

Dcluster3 1250 ***gcggtg***TTACTATAGTGACCAC***cacagtg***agacatggggca***gcaaaccct*** 1299

Dcluster2 1291 GCCAACCGTATGGCCAACCAGCCCCCACGGCCGGGCCTCACGGCCTGAGC 1340

||||||||||||||||||||||||||||||||||||||||||||||||||

Dcluster3 1300 GCCAACCGTATGGCCAACCAGCCCCCACGGCCGGGCCTCACGGCCTGAGC 1349

Dcluster2 1341 TTCAGAGACCAGACCTTCGGCCTTCTGGGCCACTCTGGGCGGATGTGGCC 1390

||||||||||||||||||||||.||||||||||||||||||||||.||||

Dcluster3 1350 TTCAGAGACCAGACCTTCGGCCATCTGGGCCACTCTGGGCGGATGCGGCC 1399

Dcluster2 1391 CGCGGGCCCGTATCCAGACTCTGAGATGACTGCACTGACCCTCCAGCCAC 1440

|.|||||||.||||||||||||||||||||||||||||||||||||||||

Dcluster3 1400 CACGGGCCCATATCCAGACTCTGAGATGACTGCACTGACCCTCCAGCCAC 1449

Dcluster2 1441 ACGTCCTAGCCCAGCAAGCTGGCCCTGACCACTAGCACTCAGTACACCCC 1490

||.|||||||||||||||||||||||||||||||||||||||||||||||

Dcluster3 1450 ACATCCTAGCCCAGCAAGCTGGCCCTGACCACTAGCACTCAGTACACCCC 1499

Dcluster2 1491 CAAACAGGGTCAGGAAGGACAGAGCCCACGCTGGGCAAACCGTCAGGTCA 1540

||||||||||||||||||||||||||||||||||||||||||||||||||

Dcluster3 1500 CAAACAGGGTCAGGAAGGACAGAGCCCACGCTGGGCAAACCGTCAGGTCA 1549

Dcluster2 1541 AGTTTCCCAGGGGCAGGACCCGAGACACCTCCAACCCTGCTGGGAGCTCA 1590

||||||||||||||||||||.|||||||||||||||||||||||||||||

Dcluster3 1550 AGTTTCCCAGGGGCAGGACCTGAGACACCTCCAACCCTGCTGGGAGCTCA 1599

Dcluster2 1591 GGGAAGCTCACAGCAGTGGACGCTGGAGCTGGGCCTGTACTCAACTGCAT 1640

||||||||||||||||||||||||||||||||||| ||||||||||||||

Dcluster3 1600 GGGAAGCTCACAGCAGTGGACGCTGGAGCTGGGCC-GTACTCAACTGCAT 1648

Dcluster2 1641 AGGAGCTCACCAGGGAGCAGGCTGGGAGGGGTTCATGCAGAGGGAGCAGC 1690

||||||||.||||||||||||||||||||||||||||||||||.||||||

Dcluster3 1649 AGGAGCTCGCCAGGGAGCAGGCTGGGAGGGGTTCATGCAGAGGCAGCAGC 1698

Dcluster2 1691 TCCCAGCATGCTGCCAGACCCGAGGTAGGGGAGTCCGGATCCAGGCCCGG 1740

||||||||||||||||||||||||||||||||||||||||||||||||||

Dcluster3 1699 TCCCAGCATGCTGCCAGACCCGAGGTAGGGGAGTCCGGATCCAGGCCCGG 1748

Dcluster2 1741 CCTGAAGGGGCTCGGGGGGCCTCTCACATGTAGCAGGGGTGTGCAGGTCC 1790

||||||||||||||||||||||||||||||||||||||||||||||||||

Dcluster3 1749 CCTGAAGGGGCTCGGGGGGCCTCTCACATGTAGCAGGGGTGTGCAGGTCC 1798

Dcluster2 1791 CTTGTGCAGTGTCCAGAGATCATGGACAGGGCGGGCACCGCACCAGAAGC 1840

||||||||||||||||||||||||||||||||||||||.|||||||||||

Dcluster3 1799 CTTGTGCAGTGTCCAGAGATCATGGACAGGGCGGGCACTGCACCAGAAGC 1848

Dcluster2 1841 CAAGCCAGTGACCAGCCTCTGCAGGAAGCACCCATGGGAGCCAACACCTA 1890

|||||||||||||||||||||||||||||.|||.||||||||||||||||

Dcluster3 1849 CAAGCCAGTGACCAGCCTCTGCAGGAAGCGCCCGTGGGAGCCAACACCTA 1898

Dcluster2 1891 AGTCTCCCTCCCAGCCCTGCAGCGTGGCCACCCACCACCCCACAGGTCAG 1940

||||||||||||||||||||||||||||||||||||||||||||||||||

Dcluster3 1899 AGTCTCCCTCCCAGCCCTGCAGCGTGGCCACCCACCACCCCACAGGTCAG 1948

Dcluster2 1941 CCTGGGGAGCTCAAGACGGACTGGCGTCCCTACCTGCCAGGTTCCTGTGC 1990

||||||||||||||||||||||||||||||||||||||||||||||||||

Dcluster3 1949 CCTGGGGAGCTCAAGACGGACTGGCGTCCCTACCTGCCAGGTTCCTGTGC 1998

Dcluster2 1991 AGGATGCTGTCCACGGCCGCAATGCTTTGGCCTCTGCTCAGCTTCTGAGG 2040

||||.|||||||||.|.||||||||||.|||||||||||||||||.||||

Dcluster3 1999 AGGACGCTGTCCACAGGCGCAATGCTTGGGCCTCTGCTCAGCTTCCGAGG 2048

Dcluster2 2041 TTCTCCGGCCCACCACCACTGACCTGAATGGGCTTGGAGTGCAGGCCCAG 2090

|||.|.|||||||||||||.||||||||.|||||.|||||||||||||||

Dcluster3 2049 TTCCCTGGCCCACCACCACCGACCTGAAGGGGCTCGGAGTGCAGGCCCAG 2098

Dcluster2 2091 CTGAACATCAGACCTGGGCTCAGAGAGCCACTCGGTCCAGGGTGCACTCA 2140

|.|.||||.|||||||||||||||.|||||||||||||||||||||||||

Dcluster3 2099 CAGGACATAAGACCTGGGCTCAGATAGCCACTCGGTCCAGGGTGCACTCA 2148

Dcluster2 2141 GCCGTGCACCTGGAGGAGGGTCCTCCATG-CCGCAGGGTTGGCTGGGCAA 2189

|||.||||||||||||.|||||||||||| ||||||..||||||||||||

Dcluster3 2149 GCCCTGCACCTGGAGGTGGGTCCTCCATGGCCGCAGCATTGGCTGGGCAA 2198

Dcluster2 2190 GGAGGCATCACAGACCACCCTGCTGTTGGGACGTCCAGCCCTGGACCAGG 2239

|||||||||.|||.|||||||||.||||||.|||||||||||||||||||

Dcluster3 2199 GGAGGCATCCCAGGCCACCCTGCCGTTGGGCCGTCCAGCCCTGGACCAGG 2248

Dcluster2 2240 ACTAGGTTCTCAGAATCCTCCCCACCACGGGACCTGTCCATACCTGGACC 2289

|||||||||||||||.||||||||||||||||||.|||||.||||||.||

Dcluster3 2249 ACTAGGTTCTCAGAACCCTCCCCACCACGGGACCCGTCCAGACCTGGGCC 2298

Dcluster2 2290 AGCTGACAGCTCCCAAGGGCTCCA-ACCCAGCAGACAAGACCAAGGG--C 2336

|||||||||||||||||||||||| |||||||||||||||||||||| |

Dcluster3 2299 AGCTGACAGCTCCCAAGGGCTCCACACCCAGCAGACAAGACCAAGGGGGC 2348

Dcluster2 2337 GCTGAAGGCGAGCAGCTAG***ggtttctga***tgccggctgtgt***tgtggtg***ATG 2386

||||||..|.|.||.||.||||||||||||||||||||||...||||.|.

Dcluster3 2349 GCTGAAAACCAACAACTGG***ggtttctga***tgccggctgtgt***cacggtg***GTA 2398

Dcluster2 2387 ATACGATAGGTGTGGTTGTAGTTATTGTAGTGTTGCTAC***cacagtg***atgc 2436

.|..|.|||.|.|.||.|||||||||. ||.|||||||||||..|

Dcluster3 2399 TTGTGGTAGCTATTGTGGTAGTTATTA------TGGTAC***cacagtg***acac 2442

Dcluster2 2437 tctcagtg***tcagaaacc***CCTGCCCCGAGGTCCCAGACCAGGGAGCCTGTC 2486

|.||...|.||||||||||.|.|||.||||||||||||||||||||||||

Dcluster3 2443 tgtccagg***acagaaacc***CCCGTCCCAAGGTCCCAGACCAGGGAGCCTGTC 2492

Dcluster2 2487 CTCCAGGTACGGGGCACCGCCCTGCCCTCGTGACCATCAGACTGCACCCT 2536

.||||.|..|||||.||||||||||||||.||||||||||||||||||||

Dcluster3 2493 TTCCAAGACCGGGGTACCGCCCTGCCCTCCTGACCATCAGACTGCACCCT 2542

Dcluster2 2537 GCACGCCCTCACCCTGCTGTCTCAGGGCAGCAACAGCCTCTGGAGGAGTC 2586

||||||||||||||..|||.|||||||.|||||||||||..|||.|.|||

Dcluster3 2543 GCACGCCCTCACCCCACTGGCTCAGGGAAGCAACAGCCTGAGGAAGGGTC 2592

Dcluster2 2587 CTGGGCCAGGCACTCGGCTGTGGAGTCCCAGACAATGATAGCCTCAGCAG 2636

|||||||||||.||.||||.|||.|.||||||||||||.|.||.||||||

Dcluster3 2593 CTGGGCCAGGCGCTGGGCTATGGGGACCCAGACAATGAGACCCCCAGCAG 2642

Dcluster2 2637 TTTCCAGCCACCTGACCCTGGAGGAGCTTGTGCAAGCTGGAGGCCGGCTG 2686

.||||||||.|||||||||||||||.|||....|.||..|.|.|.|||||

Dcluster3 2643 GTTCCAGCCCCCTGACCCTGGAGGAACTTCCAGAGGCCTGTGCCAGGCTG 2692

Dcluster2 2687 GGGCCCGGAGCCTCAGAACCCTCCCTGCCAGGGGGCCCGTCCAGACCTGG 2736

|||.||| |.|||.|||||||||||. |.|..||||||.||||||

Dcluster3 2693 GGGACCG-ACCCTAAGAACCCTCCCC-CGACAGGGCCCATCCAGA----- 2735

Dcluster2 2737 ACCAGCTGACAGCTCCCGAGGACTGCACGCCCACCAGACGAGACCAAGGG 2786

||||||.|||||.||.||.|.|||||||||.||||||||||

Dcluster3 2736 ---------CAGCTCTCGAGGTCTCCATGTCCACCAGACAAGACCAAGGG 2776

Dcluster2 2787 GGCGCTGAAGGCCAGCAGCTGG***ggtttctga*tgccggctgtgt*cacggtg*** 2836

|||||||||||||||||.|||||||||.||||||||||||||||||||||

Dcluster3 2777 GGCGCTGAAGGCCAGCAACTGG***ggtttttga***tgccggctgtgt***cacggtg*** 2826

Dcluster2 2837 **GTAGTTGTCCTGATGGTTATAGTTATGGTTATGGTTGTGGTTATGG**TTAT 2886

||||||

Dcluster3 2827 GTAGTT-------------------------------------------- 2832

Dcluster2 2887 **GGTTGTAGTGGTTATGATTGTTATGGTTATGGTGGTTATGGTGGTTATGG** 2936

|||.|.|||||||||||||||||||||||.|||||||

Dcluster3 2833 -------------ATGGTGGTTATGGTTATGGTGGTTATGGTTGTTATGG 2869

Dcluster2 2937 **TGGTTATGGTTATAGTAGTTATAGTTATAGTTATACTTACGAATATAC*ca*** 2986

| |||.|||||..|||.|..|||||||

Dcluster3 2870 T------------------------TATGGTTATGGTTATGGTTATAC***ca*** 2895

Dcluster2 2987 ***cagtg***atactctctggg**acaaaaacc**CCTGCCCCTGAGGGTCCACGGCCA 3036

||||||||||||||||||||||||||||||||||||||||||||||.|||

Dcluster3 2896 ***cagtg***atactctctggg***acaaaaacc***CCTGCCCCTGAGGGTCCACGACCA 2945

Dcluster2 3037 GGGATCCTGGAGGCTGTGTTTGGGGCACCGCCCTGCCCTCCTGCCATTGG 3086

||||..|||.|.|||||||||||||||||.||||||||||||||||||||

Dcluster3 2946 GGGAATCTGTAAGCTGTGTTTGGGGCACCACCCTGCCCTCCTGCCATTGG 2995

Dcluster2 3087 ACTACAACCTGCACGCCCTCACCCTGCTGACTCGGGCAGCAACAGCCTAA 3136

|||.||.|||||||||||||||||||||||||||||||||||||||||||

Dcluster3 2996 ACTGCACCCTGCACGCCCTCACCCTGCTGACTCGGGCAGCAACAGCCTAA 3045

Dcluster2 3137 GATAAGAGTCCTGGGCCAGGCGCTGGGCTGTGGATACCCAGACAATGAGA 3186

||||||.|||||||||||||.|||||||||||||||||||||||||||||

Dcluster3 3046 GATAAGGGTCCTGGGCCAGGAGCTGGGCTGTGGATACCCAGACAATGAGA 3095

Dcluster2 3187 TCTCCAGCAGGTTCCCACCTCCTGACCCTGGAGGAACTTCCGCAGCCCTG 3236

||||||||||||||||.||||||||.| ||||||||||.||||||.||||

Dcluster3 3096 TCTCCAGCAGGTTCCCGCCTCCTGATC-TGGAGGAACTGCCGCAGGCCTG 3144

Dcluster2 3237 TGGCAGGCTGGGGACCGACCCTAAGAACCCTCCCCCC------------- 3273

||.||||||||||||||||||||||||||||||||||

Dcluster3 3145 TGCCAGGCTGGGGACCGACCCTAAGAACCCTCCCCCCCGCAGGGCCATCC 3194

Dcluster2 3274 -------------------------------------------------- 3273

Dcluster3 3195 AGACCTGGGCCAGCTGACAGCTCCCGAGGACTCCACGTCCACCAGACGAG 3244

Dcluster2 3274 -------------------------------------------------- 3273

Dcluster3 3245 ACCAAGGGGACGCCGAAGGCCAGCAGCTGG***ggtttctga***tgccggctgtg 3294

Dcluster2 3274 -------------------------------------------------- 3273

Dcluster3 3295 t***tgtggtg***ATGATACGATAGGTGTGGTTTTAGTTATTGTAGTGTTGCTAC 3344

Dcluster2 3274 -------------------------------------------------- 3273

Dcluster3 3345 ***cacagtg***acgctctcagtg***tcagaaacc***CCTGCCCCGAGGTCCCAGACCA 3394

Dcluster2 3274 -------------------------------------------------- 3273

Dcluster3 3395 GGGAGCTTGTCCTCCAGGTCCGGGGTACGGCCCTGCCCTCCTGACCATCG 3444

Dcluster2 3274 -------------------------------------------------- 3273

Dcluster3 3445 GACTGCACCCTGCATGCCCTCACCCTGCTGACTCAGGGCAGCAACAGAAT 3494

Dcluster2 3274 -------------------------------------------------- 3273

Dcluster3 3495 GAGGAAGGGTCCTGGGCCAAGAGCTTGGCTGCTGGGACCCAGACAATGAG 3544

Dcluster2 3274 -------------------------------------------------- 3273

Dcluster3 3545 GCCCCCAGCAAGTTCCAGCTCCCTGACCCTGGAGGAGGTTGTGCAGGTCA 3594

Dcluster2 3274 ----------------------------------------GCAGGGCCCG 3283

||||||||||

Dcluster3 3595 GAGGCCGGCTGGGGACCGGATCCTCAGAACCCTCCCTGCCGCAGGGCCCG 3644

Dcluster2 3284 TCCAGACCTGAACCAGCTGACAGCTCCCGAGGGCTCCACGCCCCCCAGAC 3333

||||||| ||.|||||||||||||||||.|||.||||||||||.||||||

Dcluster3 3645 TCCAGAC-TGGACCAGCTGACAGCTCCCAAGGACTCCACGCCCACCAGAC 3693

Dcluster2 3334 GAGACCAAGGGGGCGCCGAAGGCCAGCAGCTGG***ggtttctga***tgccggct 3383

||||||||||||||||||||||||||||||.|||||||||||||||.|||

Dcluster3 3694 GAGACCAAGGGGGCGCCGAAGGCCAGCAGCCGG***ggtttctga***tgccagct 3743

Dcluster2 3384 gtgt***cacggtg***GTAGTTGTTATAGTGGTTATGGTTATGGTTGTGGTTATG 3433

|||||||||||||||||||||||||||||||||||||||||.|||||.||

Dcluster3 3744 gtgt***cacggtg***GTAGTTGTTATAGTGGTTATGGTTATGGTTATGGTTGTG 3793

Dcluster2 3434 GTTATGGTTATGATTATAC***cacagtg***acactctctggg**acaaaaacc**  3480

||||||||||||.||||||||||||||||||||||||||||||||||

Dcluster3 3794 GTTATGGTTATGGTTATAC***cacagtg***acactctctggg***acaaaaacc*** 3840
