# Supplementary material for: Formation of ultralong DH regions through genomic rearrangement
Source: BMC Immunol. 2020 Jun 2;21:30. doi: 10.1186/s12865-020-00359-8 (PMC7265228; doi:10.1186/s12865-020-00359-8)
Supplement: Supplementary file 3 — Additional file 3: Figure S3. Phylogenetic of DH paralogs from clusters 2 and 3. [file 12865_2020_359_MOESM3_ESM.docx]

**Supplemental Figure 3.** Homology of IGHD regions from clusters 2 and 3. The DH regions IGHD1-2, IGHD2-2, IGHD5-2, IGHD8-2, and IGHD6-2 from cluster 2 and IGHD1-3, IGHD2-3, IGHD3-3, IGHD7-3, IGHD5-3, and IGHD6-3 from cluster 3 were aligned using Clustal2.1 and phylogenetic tree (A) and percent identity matrix (B) shown. The IGHD3-3 or IGHD7-3 regions do not have clear paralogs on cluster 2. Paralogs on the two clusters are highlighted green. Percent identities above 95% are in bold.

**A.**


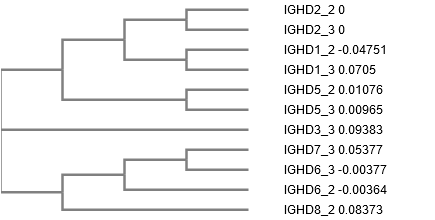


**B.**

# Percent Identity Matrix - created by Clustal2.1

#

#

**2-2 2-3 1-2 1-3 5-2 5-3 3-3 7-3 8-2 6-2 6-3**

1: IGHD2_2 100.00 **100.00** 43.75 36.11 48.61 47.22 45.83 48.61 48.61 50.00 48.33

2: IGHD2_3 **100.00** 100.00 43.75 36.11 48.61 47.22 45.83 48.61 48.61 50.00 48.33

3: IGHD1_2 43.75 43.75 100.00 **97.70** 55.17 55.17 58.06 44.12 47.06 60.00 57.78

4: IGHD1_3 36.11 36.11 **97.70** 100.00 39.80 40.82 43.48 32.79 40.46 47.19 46.07

5: IGHD5_2 48.61 48.61 55.17 39.80 100.00 **97.96** 71.11 75.29 72.45 78.08 76.71

6: IGHD5_3 47.22 47.22 55.17 40.82 **97.96** 100.00 72.22 74.12 71.43 79.45 78.08

7: IGHD3_3 45.83 45.83 58.06 43.48 71.11 72.22 100.00 76.62 80.43 88.00 85.33

8: IGHD7_3 48.61 48.61 44.12 32.79 75.29 74.12 76.62 100.00 81.30 **95.00** **95.00**

9: IGHD8_2 48.61 48.61 47.06 40.46 72.45 71.43 80.43 81.30 100.00 89.47 89.47

10: IGHD6_2 50.00 50.00 60.00 47.19 78.08 79.45 88.00 **95.00** 89.47 100.00 **96.49**

11: IGHD6_3 48.33 48.33 57.78 46.07 76.71 78.08 85.33 **95.00** 89.47 **96.49** 100.00
