# Supplementary material for: Formation of ultralong DH regions through genomic rearrangement
Source: BMC Immunol. 2020 Jun 2;21:30. doi: 10.1186/s12865-020-00359-8 (PMC7265228; doi:10.1186/s12865-020-00359-8)
Supplement: Supplementary file 4 — Additional file 4: Figure S4. Phylogenetic analysis of intergenic regions from clusters 2 and 3. [file 12865_2020_359_MOESM4_ESM.docx]

**Supplemental Figure 4.** Homology of intergenic regions. The intergenic regions between IGHD2-2 and IGHD5-2 (before5-2), IGHD5-2 and IGHD8-2 (before8-2), IGHD8-2 and IGHD6-2 (before6-2) of cluster 2 and IGHD2-3 and IGHD3-3 (before3-3), IGHD3-3 and IGHD7-3 (before7-3), IGHD7-3 and IGHD5-3 (before 5-3), and IGHD5-3 and IGHD6-3 (before6-3) of cluster 3 were aligned using Clustal2.1 and phylogenetic tree (A) and percent identity matrix (B) shown. The region between IGHD3-3 and IGHD7-3 (before7-3) does not have a clear paralog on cluster 3. Paralogs on the two clusters are highlighted green.

**A.**


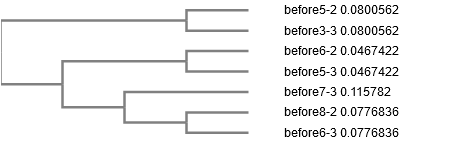


**B.**

# Percent Identity Matrix - created by Clustal2.1

#

#

**b5-2 b3-3 b6-2 b5-3 b7-3 b8-2 b6-3**

1: before5-2 100.00 85.23 44.17 42.46 43.27 48.78 49.85

2: before3-3 85.23 100.00 52.45 51.38 51.92 57.32 57.19

3: before6-2 44.17 52.45 100.00 92.35 76.19 74.15 73.50

4: before5-3 42.46 51.38 92.35 100.00 77.61 73.22 72.57

5: before7-3 43.27 51.92 76.19 77.61 100.00 80.53 80.24

6: before8-2 48.78 57.32 74.15 73.22 80.53 100.00 87.29

7: before6-3 49.85 57.19 73.50 72.57 80.24 87.29 100.00
