# Supplementary material for: Formation of ultralong DH regions through genomic rearrangement
Source: BMC Immunol. 2020 Jun 2;21:30. doi: 10.1186/s12865-020-00359-8 (PMC7265228; doi:10.1186/s12865-020-00359-8)
Supplement: Supplementary file 5 — Additional file 5: Figure S5. Alignment of IGHD7 family members with IGHD8–2. [file 12865_2020_359_MOESM5_ESM.docx]

**Supplemental Figure 5.** Local alignment of IGHD7-3 and IGHD7-4 with IGHD8-2.

########################################

# Program: matcher

# Rundate: Sat 5 Oct 2019 00:28:58

# Commandline: matcher

# -auto

# -stdout

# -asequence emboss_matcher-I20191005-002855-0888-87948780-p2m.asequence

# -bsequence emboss_matcher-I20191005-002855-0888-87948780-p2m.bsequence

# -datafile EDNAFULL

# -gapopen 16

# -gapextend 4

# -alternatives 1

# -aformat3 pair

# -snucleotide1

# -snucleotide2

# Align_format: pair

# Report_file: stdout

########################################

#=======================================

#

# Aligned_sequences: 2

# 1: IGHD8_2

# 2: IGHD7_3

# Matrix: EDNAFULL

# Gap_penalty: 16

# Extend_penalty: 4

#

# Length: 97

# Identity: 83/97 (85.6%)

# Similarity: 83/97 (85.6%)

# Gaps: 0/97 ( 0.0%)

# Score: 359

#

#

#=======================================

IGHD8_2 108 TGGTGGTTATGGTGGTTATGGTGGTTATGGTTATAGTAGTTATAGTTATA 157

||||.|||||||||||||||||..|..|||||||.||.|||||.|||||.

IGHD7_3 27 TGGTAGTTATGGTGGTTATGGTTATGGTGGTTATGGTTGTTATGGTTATG 76

IGHD8_2 158 GTTATACTTACGAATATACCACAGTGATACTCTCTGGGACAAAAACC 204

|||||..|||.|..|||||||||||||||||||||||||||||||||

IGHD7_3 77 GTTATGGTTATGGTTATACCACAGTGATACTCTCTGGGACAAAAACC 123

#---------------------------------------

#---------------------------------------

########################################

# Program: matcher

# Rundate: Sat 5 Oct 2019 00:30:17

# Commandline: matcher

# -auto

# -stdout

# -asequence emboss_matcher-I20191005-003015-0194-38128626-p2m.asequence

# -bsequence emboss_matcher-I20191005-003015-0194-38128626-p2m.bsequence

# -datafile EDNAFULL

# -gapopen 16

# -gapextend 4

# -alternatives 1

# -aformat3 pair

# -snucleotide1

# -snucleotide2

# Align_format: pair

# Report_file: stdout

########################################

#=======================================

#

# Aligned_sequences: 2

# 1: IGHD8_2

# 2: IGHD7_4

# Matrix: EDNAFULL

# Gap_penalty: 16

# Extend_penalty: 4

#

# Length: 132

# Identity: 102/132 (77.3%)

# Similarity: 102/132 (77.3%)

# Gaps: 3/132 ( 2.3%)

# Score: 378

#

#

#=======================================

IGHD8_2 73 GGTTATGGTTGTAGTGGTTATGATTGTTATGGTTATGGTGGTTATGGTGG 122

||||.|.|.||..|....|.|.|..||..|.||||||||||||||| |

IGHD7_4 1 GGTTTTTGATGCCGGCTGTGTCACGGTGGTAGTTATGGTGGTTATG---G 47

IGHD8_2 123 TTATGGTGGTTATGGTTATAGTAGTTATAGTTATAGTTATACTTACGAAT 172

|||||||||||||||||.|..|.|||||.|||||.|||||..|||.|..|

IGHD7_4 48 TTATGGTGGTTATGGTTGTTATGGTTATGGTTATGGTTATGGTTATGGTT 97

IGHD8_2 173 ATACCACAGTGATACTCTCTGGGACAAAAACC 204

||||||||||||||||||||||||||||||||

IGHD7_4 98 ATACCACAGTGATACTCTCTGGGACAAAAACC 129

#---------------------------------------

#---------------------------------------
