# Supplementary material for: Formation of ultralong DH regions through genomic rearrangement
Source: BMC Immunol. 2020 Jun 2;21:30. doi: 10.1186/s12865-020-00359-8 (PMC7265228; doi:10.1186/s12865-020-00359-8)
Supplement: Supplementary file 6 — Additional file 6: Figure S6. Identification ultralong IGHD region homologs in Bos taurus, Bos grunniens and Bison bison. [file 12865_2020_359_MOESM6_ESM.docx]

**Supplemental Figure 6.** Ultralong DH region variants differ in in length. A multiple sequence alignment was performed using MUSCLE with the two *Bos taurus* IGHD8-2 polymorphisms, as well as IGHD8-2 homologs from *Bison bison* and *Bos grunniens*. The RSS are in lowercase, with heptamer and nonamer underlined and in italics.

CLUSTAL multiple sequence alignment by MUSCLE (3.8)

IGHD8-2*01 *ggtttctga*tgccggctgtgt*cacggtg*GTAGTTGTCCTGATGGTTATAGTTAT------

IGHD8-2*02 *ggtttctga*tgccggctgtgt*cacggtg*GTAGTTGTCCTGATGGTTATAGTTAT------

Bison_IGHD8-2 *ggtttctga*tgccggctgtgt*cacggtg*GTAGTTGTCCTGATGGTTATAGTTATGGTTAT

BosGru_IGHD8-2 *ggtttctga*tgccggctgtgt*cacggtg*GTAGTTGTCCTGATGGTTATGGTTATGGTTAT

************************************************ *****

IGHD8-2*01 GGTTATGGTTGTGGTTATG---GTTATGGTTGTAGTGGTTATGATTGTTATGGTTATGGT

IGHD8-2*02 GGTTATGGTTGTGGTTATGGTAGTTATGGTTGTAGTGGTTATGATTGTTATGGTTATGGT

Bison_IGHD8-2 GGTTATGGTTGTGGTTATGGTGGTTATGGTTGTAGTGGTTATGATTGTTATGGTTATGGT

BosGru_IGHD8-2 GGTTATGGTTGTGGTTATGGTGGTTATGGTTGTAGTGGTTATGATTGTTATGGTTATGGT

******************* **************************************

IGHD8-2*01 GGTTATGGTGGTTATGGTGGTTA---TGGTTATAGTAGTTATAGTTATAGTTATACTTAC

IGHD8-2*02 GGTTATGGTGGTTATGGTGGTTATGGTGGTTATAGTAGTTATAGTTATAGTTATAGTTAC

Bison_IGHD8-2 AGTTA---------------------TGGTTATAGTGGTTATAGTTATAGTTATAGTTAC

BosGru_IGHD8-2 GGTTA---------------------TGGTTATAGTGGTTATAGTTATAGTTATAGTTAC

**** ********** ****************** ****

IGHD8-2*01 GAATATAC*cacagtg*atactctctggg*acaaaaacc*

IGHD8-2*02 GAATATAC*cacagtg*atactctctggg*acaaaaacc*

Bison_IGHD8-2 GAATATAC*cacagtg*atactctctggg*acaaaaacc*

BosGru_IGHD8-2 GAATATAC*cacagtg*atactctctggg*acaaaaacc*

************************************
