# Supplementary material for: Formation of ultralong DH regions through genomic rearrangement
Source: BMC Immunol. 2020 Jun 2;21:30. doi: 10.1186/s12865-020-00359-8 (PMC7265228; doi:10.1186/s12865-020-00359-8)
Supplement: Supplementary file 7 — Additional file 7: Figure S7. Alignment of IGHD3 and IGHD6 family members with IGHD8–2. [file 12865_2020_359_MOESM7_ESM.docx]

**Supplemental Figure 7.** Alignment of IGHD3 and IGHD6 family members with IGHD8-2.

########################################

# Program: matcher

# Rundate: Sat 5 Oct 2019 00:41:09

# Commandline: matcher

# -auto

# -stdout

# -asequence emboss_matcher-I20191005-004107-0321-40057744-p1m.asequence

# -bsequence emboss_matcher-I20191005-004107-0321-40057744-p1m.bsequence

# -datafile EDNAFULL

# -gapopen 16

# -gapextend 4

# -alternatives 1

# -aformat3 pair

# -snucleotide1

# -snucleotide2

# Align_format: pair

# Report_file: stdout

########################################

#=======================================

#

# Aligned_sequences: 2

# 1: IGHD8_2

# 2: IGHD3_4

# Matrix: EDNAFULL

# Gap_penalty: 16

# Extend_penalty: 4

#

# Length: 65

# Identity: 52/65 (80.0%)

# Similarity: 52/65 (80.0%)

# Gaps: 5/65 ( 7.7%)

# Score: 184

#

#

#=======================================

IGHD8_2 1 GGTTTCTGATGCCGGCTGTGTCACGGTGGTA--GTTGTCCTGATGGTTAT 48

||||||||||||||||||||||||||||||| ||.||....||.||..|

IGHD3_4 1 GGTTTCTGATGCCGGCTGTGTCACGGTGGTATTGTGGTAGCTATTGTGGT 50

IGHD8_2 49 AGTTATGGTTATGGT 63

|||||| ||||||

IGHD3_4 51 AGTTAT---TATGGT 62

#---------------------------------------

#---------------------------------------

########################################

# Program: matcher

# Rundate: Sat 5 Oct 2019 00:42:25

# Commandline: matcher

# -auto

# -stdout

# -asequence emboss_matcher-I20191005-004222-0822-86095519-p2m.asequence

# -bsequence emboss_matcher-I20191005-004222-0822-86095519-p2m.bsequence

# -datafile EDNAFULL

# -gapopen 16

# -gapextend 4

# -alternatives 1

# -aformat3 pair

# -snucleotide1

# -snucleotide2

# Align_format: pair

# Report_file: stdout

########################################

#=======================================

#

# Aligned_sequences: 2

# 1: IGHD8_2

# 2: IGHD3_3

# Matrix: EDNAFULL

# Gap_penalty: 16

# Extend_penalty: 4

#

# Length: 65

# Identity: 52/65 (80.0%)

# Similarity: 52/65 (80.0%)

# Gaps: 5/65 ( 7.7%)

# Score: 184

#

#

#=======================================

IGHD8_2 1 GGTTTCTGATGCCGGCTGTGTCACGGTGGTA--GTTGTCCTGATGGTTAT 48

||||||||||||||||||||||||||||||| ||.||....||.||..|

IGHD3_3 1 GGTTTCTGATGCCGGCTGTGTCACGGTGGTATTGTGGTAGCTATTGTGGT 50

IGHD8_2 49 AGTTATGGTTATGGT 63

|||||| ||||||

IGHD3_3 51 AGTTAT---TATGGT 62

#---------------------------------------

#---------------------------------------

########################################

# Program: matcher

# Rundate: Fri 4 Oct 2019 23:30:39

# Commandline: matcher

# -auto

# -stdout

# -asequence emboss_matcher-I20191004-233038-0324-2606874-p2m.asequence

# -bsequence emboss_matcher-I20191004-233038-0324-2606874-p2m.bsequence

# -datafile EDNAFULL

# -gapopen 16

# -gapextend 4

# -alternatives 1

# -aformat3 pair

# -snucleotide1

# -snucleotide2

# Align_format: pair

# Report_file: stdout

########################################

#=======================================

#

# Aligned_sequences: 2

# 1: IGHD8_2

# 2: IGHD6_2

# Matrix: EDNAFULL

# Gap_penalty: 16

# Extend_penalty: 4

#

# Length: 85

# Identity: 76/85 (89.4%)

# Similarity: 76/85 (89.4%)

# Gaps: 0/85 ( 0.0%)

# Score: 344

#

#

#=======================================

IGHD8_2 1 GGTTTCTGATGCCGGCTGTGTCACGGTGGTAGTTGTCCTGATGGTTATAG 50

||||||||||||||||||||||||||||||||||||..|..|||||||.|

IGHD6_2 1 GGTTTCTGATGCCGGCTGTGTCACGGTGGTAGTTGTTATAGTGGTTATGG 50

IGHD8_2 51 TTATGGTTATGGTTGTGGTTATGGTTATGGTTGTA 85

||||||||.|||||.||||||||||||||.||.||

IGHD6_2 51 TTATGGTTGTGGTTATGGTTATGGTTATGATTATA 85

#---------------------------------------

#---------------------------------------

########################################

# Program: matcher

# Rundate: Fri 4 Oct 2019 23:33:07

# Commandline: matcher

# -auto

# -stdout

# -asequence emboss_matcher-I20191004-233305-0188-59118204-p1m.asequence

# -bsequence emboss_matcher-I20191004-233305-0188-59118204-p1m.bsequence

# -datafile EDNAFULL

# -gapopen 16

# -gapextend 4

# -alternatives 1

# -aformat3 pair

# -snucleotide1

# -snucleotide2

# Align_format: pair

# Report_file: stdout

########################################

#=======================================

#

# Aligned_sequences: 2

# 1: IGHD8_2

# 2: IGHD6_3

# Matrix: EDNAFULL

# Gap_penalty: 16

# Extend_penalty: 4

#

# Length: 85

# Identity: 78/85 (91.8%)

# Similarity: 78/85 (91.8%)

# Gaps: 0/85 ( 0.0%)

# Score: 362

#

#

#=======================================

IGHD8_2 1 GGTTTCTGATGCCGGCTGTGTCACGGTGGTAGTTGTCCTGATGGTTATAG 50

|||||||||||||.||||||||||||||||||||||..|..|||||||.|

IGHD6_3 1 GGTTTCTGATGCCAGCTGTGTCACGGTGGTAGTTGTTATAGTGGTTATGG 50

IGHD8_2 51 TTATGGTTATGGTTGTGGTTATGGTTATGGTTGTA 85

||||||||||||||||||||||||||||||||.||

IGHD6_3 51 TTATGGTTATGGTTGTGGTTATGGTTATGGTTATA 85

#---------------------------------------

#---------------------------------------
